# Development of BioPolyurethane Coatings from Biomass-Derived Alkylphenol Polyols—A Green Alternative

**DOI:** 10.3390/polym15112561

**Published:** 2023-06-02

**Authors:** Tiago A. R. Silva, Ana C. Marques, Rui G. dos Santos, Rana A. Shakoor, Maryna Taryba, Maria Fátima Montemor

**Affiliations:** 1 Centro de Química Estrutural (CQE), Institute of Molecular Sciences (IMS), Departamento de Engenharia Química (DEQ), Instituto Superior Técnico (IST), Universidade de Lisboa, Av. Rovisco Pais 1, 1049-001 Lisboa, Portugal; maryna.taryba@tecnico.ulisboa.pt (M.T.); mfmontemor@tecnico.ulisboa.pt (M.F.M.); 2 Centro de Recursos Naturais e Ambiente (CERENA), Departamento de Engenharia Química (DEQ), Instituto Superior Técnico, Universidade de Lisboa, Av. Rovisco Pais 1, 1049-001 Lisboa, Portugal; rui.galhano@tecnico.ulisboa.pt; 3 Centre for Advanced Materials (CAM), Qatar University, 9FHQ + JMF, Doha P.O. Box 2713, Qatar; shakoor@qu.edu.qa

**Keywords:** thermochemical liquefaction, biomass, biopolyols, biopolyurethane, polyurethane coatings, corrosion protection

## Abstract

Bio-based polyols were obtained from the thermochemical liquefaction of two biomass feedstocks, pinewood and *Stipa tenacissima*, with conversion rates varying between 71.9 and 79.3 wt.%, and comprehensively characterized. They exhibit phenolic and aliphatic moieties displaying hydroxyl (OH) functional groups, as confirmed by attenuated total reflectance-Fourier transform infrared spectroscopy (ATR-FTIR) and nuclear magnetic resonance spectroscopy (NMR) analysis. The biopolyols obtained were successfully employed as a green raw material to produce bio-based polyurethane (BioPU) coatings on carbon steel substrates, using, as an isocyanate source, a commercial bio-based polyisocyanate—Desmodur^®^ Eco N7300. The BioPU coatings were analyzed in terms of chemical structure, the extent of the reaction of the isocyanate species, thermal stability, hydrophobicity, and adhesion strength. They show moderate thermal stability at temperatures up to 100 °C, and a mild hydrophobicity, displaying contact angles between 68° and 86°. The adhesion tests reveal similar pull-off strength values (ca. 2.2 MPa) for the BioPU either prepared with pinewood and *Stipa*-derived biopolyols (BPUI and BPUII). Electrochemical impedance spectroscopy (EIS) measurements were carried out on the coated substrates for 60 days in 0.05 M NaCl solution. Good corrosion protection properties were achieved for the coatings, with particular emphasis on the coating prepared with the pinewood-derived polyol, which exhibited a low-frequency impedance modulus normalized for the coating thickness of 6.1 × 10^10^ Ω cm at the end of the 60 days test, three times higher than for coatings prepared with *Stipa*-derived biopolyols. The produced BioPU formulations show great potential for application as coatings, and for further modification with bio-based fillers and corrosion inhibitors.

## 1. Introduction

Steel and steel-made infrastructures are critical for the expansion and operation of industry and global economies. Short-term forecasts for steel demand project further growth, with the industrialization of developing nations being part of the driving force [1,2]. Steel is susceptible to corrosion failures, particularly in aggressive environments, and such failures pose a serious and expensive problem, with estimated costs reaching over 3% of global gross domestic product (GDP) [3,4,5,6]. Effective corrosion prevention and management methods can be divided into four broad categories: alteration of the environment, modification of the steel composition, adjustment of procedures, and application of protective coatings [7]. Inorganic or organic coatings are efficient approaches to protect metallic components, including steel, from the detrimental action of aggressive environments, such as chemical and mechanical damage, moisture, and biological degradation, thus preventing and contributing to the efficient management of corrosion failures [3].

**Figure 1:**
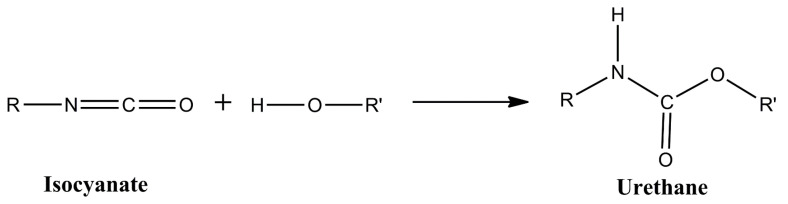
Urethane formation from the reaction of an isocyanate with a hydroxyl functional group.

Polyurethane (PU) coatings are widely used for the corrosion protection of steel. PU consists of polymer chains or networks containing urethane groups, which are formed from the reaction of an isocyanate and a hydroxyl group (Figure 1).

They are generally composed of soft and hard segments in a single polymeric matrix and are usually known to possess excellent adhesion, abrasion resistance, and toughness, along with a high corrosion protection ability and chemical resistance [8,9].

PUs are commonly produced from the step-growth polymerization of diisocyanates or polyisocyanates with glycol, or polyol compounds, without the liberation of low-molecular-weight by-products. A cross-linked polymer is formed if one or both reactants are multifunctional. The soft segments are usually relative to the polyol-derived chains, while the hard segments derive from the highly reactive isocyanate compounds. Commercial PU synthesis often uses petroleum-based raw materials, not complying with the trendy “green chemistry” and sustainability requirements, raising health and environmental concerns.

The main driving forces for the replacement of common fossil-based raw materials with bio-based feedstocks derive from the tendency for the depletion of fossil fuel feedstocks, increasing demands of energy at the global scale, and other environmental concerns, which aim at the transition to a more circular economy and the reduction of carbon footprint [6,8,10,11]. Moreover, the possibility to impart new functionalities to PU coatings also promotes the development of biopolyurethane (BioPU) chemistries, where bio-based feedstock raw materials replace common fossil-based raw materials. At present, BioPUs are being synthesized from vegetable oils obtained from various plant feedstocks such as castor, cotton, rapeseed, palm, and soybean [8,9,11,12,13,14,15,16,17], as well as from lignin and lignin derivatives [18,19,20,21,22,23,24]. Lignin and lignin derivatives are commonly obtained as subproducts of the paper and pulp industry, among other bio-renewable sources [25,26].

Plant biomass is mainly composed of organic polymers, namely, cellulose, hemicellulose, and lignin. It has the advantages of being an inexpensive raw material in addition to possessing high renewability, sustainability, and accessibility. Many processes, either thermochemical, biochemical, or mechanical, can be used to break down the biomass’ complex structure into smaller molecules and manageable fractions. Thermochemical conversion processes include pyrolysis, gasification, and liquefaction [27,28,29,30]. The thermochemical liquefaction of lignocellulosic biomass is a process that consists of the depolymerization and solubilization of biomass at moderate temperatures in a suitable solvent, usually polyhydric alcohols. The process has been widely investigated and has been shown to be a great tool to add value to biomass subproducts and wastes [27,28,31,32,33,34,35,36,37,38]. In the process of thermochemical liquefaction, lignin is degraded into phenolic compounds, while cellulose and hemicellulose are hydrolyzed into monosaccharides such as glucose and xylose, and further to acids, aldehydes, ketones [29,35]. Thermochemical liquefaction is heavily influenced by several factors, the most important being biomass composition, temperature, type of solvent, residence time, the ratio of biomass to solvent, and the presence of a catalyst [27,29,34]. When it comes to solvents, simple alcohols, such as methanol, ethanol, propanol, and butanol, give higher conversions, but have lower boiling points, requiring high-pressure reactors [27]. Longer-chain alcohols such as glycerol, ethylene glycol, 2-ethyl hexanol, or polyethylene glycol are widely used [29,34,35]. The effect of the solvent in the thermochemical liquefaction of lignocellulosic biomass has been widely studied; however, the reaction pathways and the solvent influence are many times still unclear, mostly due to the complexity and variability of the biomass and process conditions. High-polar and -protic solvents have been shown to have higher efficiency. This can be, in part, due to the products of biomass decomposition being rich in similar functional groups, allowing good solubility. The solvent capacity to act as hydrogen donor also influences the efficiency of the process. It is stated that the presence of a hydrogen donor solvent may facilitate the hydrocracking of large molecules as well as the stabilization of free radicals and molecular rearrangements [39,40]. A low mass ratio of biomass to solvent, commonly 1:3 to 1:5, contributes to high reaction yield as the products are more readily extracted to the solvent medium [32,34]. The depolymerization reactions usually occur in the presence of a catalyst, with Brønsted–Lowry acids being commonly used. Among these, p-toluenesulfonic acid has been reported to display high conversion rates without leading to secondary condensation reactions (by-product formation) and repolymerization reactions [41].

Despite the recent focus on developing BioPU from biomass feedstocks, and on the potential of thermochemical liquefaction to turn biomass subproducts and residues into polyols, relevant studies on the use of these thermochemical liquefaction polyols in BioPU coatings are relatively scarce. Most of these studies apply the liquified biomass-derived polyols to the production of PU foams [42,43,44,45,46,47,48] and adhesives [49,50,51], and are not focused on coatings, as the present paper is. Recently, Gosz et al. [52,53] developed BioPU from biopolyols derived from the thermochemical liquefaction of liquified oak and alder wood sawdust, reporting an optimum liquefaction temperature of 150 °C for a period of 6 h. It was stated that the obtained PUs possess a high Young’s module, up to 839 MPa, and beneficial properties for coating wood decks. Olszewski et al. [54] suggested that PU wood composites could be a green alternative for the construction, furniture, or automotive industries if biopolyols from the thermochemical liquefaction of wood shavings are used as raw materials. In their work, liquefaction was employed at a temperature of 150 °C for 6 h utilizing three solvents: glycerol, poly(ethylene glycol), and a 1:1 mixture of both. The obtained biopolyols were mixed with wood shavings, which were used as filler, before adding polymeric methylene diphenyl diisocyanate (pMDI). The mixture of all components was subjected to a hydraulic pressing machine. The resulting PU composite was said to possess improved characteristics, such as hardness, impact strength, flexural strength, and Young’s module.

**Figure 2:**
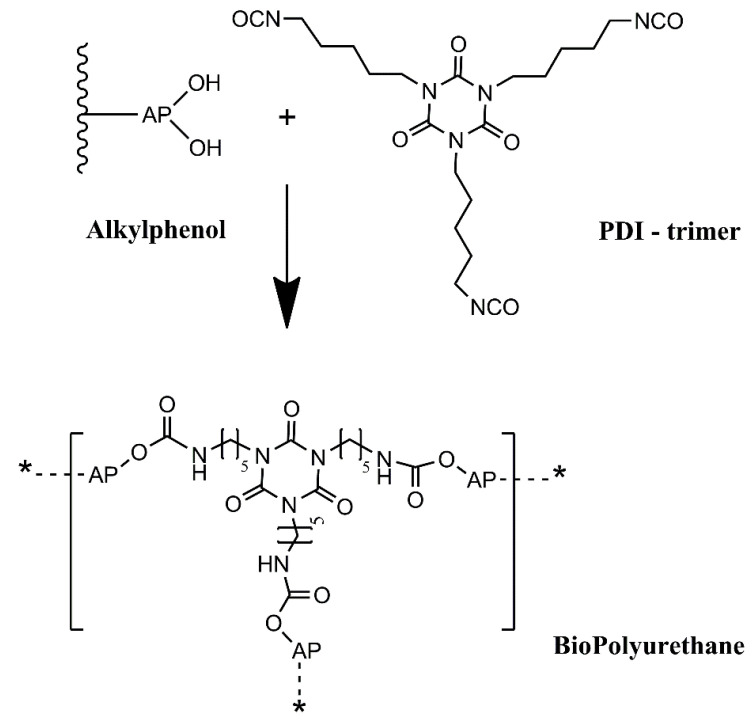
Generic scheme of the reaction between an alkylphenol polyol (AP) and a PDI trimer polyisocyanate to give a BioPU.

In this work, we intend to develop an alternative green path, where bio-based raw materials, either at the isocyanate and polyol component level, are employed to produce polyurethane coatings for corrosion protection. This will add value to biomass residues, contributing to a more circular economy, while alleviating the dependency on fossil-based raw materials. Thus, this work reports a novel BioPU-based coating for corrosion protection of steel, where petroleum-derived polyols and diisocyanates are fully replaced by bio-based feedstock raw materials, obtained from the thermochemical liquefaction of pinewood and *Stipa tenacissima* biomasses, and a new green commercially available bio-based pentamethylene diisocyanate (PDI) trimer, Desmodur^®^ Eco N7300 (Figure 2). This compound was the first commercial bio-based PDI isocyanurate trimer, launched by Covestro in 2015, in which approximately 68% of the carbon content is bio-based. The application of residues of pinewood biomass shows that they can be employed for much more than fuel for burning furnaces. At the same time, the utilization of *Stipa tenacissima* not only illustrates the flexibility of the process, as this fibrous grass nature contrasts with the wood residues utilized, but it also provides economic valorization to this biomass, which is endemic to the Mediterranean regions, south of the Iberian Peninsula and in the north of Africa. It is mainly used in the handicraft industry or for the production of paper, and currently has a low economic value. A green solvent, 2-methyl tetrahydrofuran (2-MTHF), was also employed to further promote a sustainable coating. The solvent 2-MTHF can be derived from bio-renewable sources, utilizing reagents such as furfural or levulinic acid for its synthesis. Moreover, it is reported that 2-MTHF holds high biodegradability, being abiotically degraded by sunlight and air, making it a possible alternative solvent for environmentally friendly, green synthesis processes [55,56].

The resulting BioPU base coatings reveal good anti-corrosion properties, as demonstrated by electrochemical impedance spectroscopy, and hold potential for further modification with bio-based anti-corrosion to improve the corrosion protection of metallic infrastructures.

## 2. Materials and Methods

### 2.1. Materials

Pinewood shaves purchased from Versele-Laga (Deinze, Belgium), and *Stipa tenacissima* grass (referred to as *Stipa* throughout this paper), kindly provided by a Tunisian company, were used as raw materials for the thermochemical liquefaction process. The solvents 2-ethyl hexanol (2EH; ≥90 wt.%), propylene glycol (PG; ≥90 wt.%), 2-methyl tetrahydrofuran (2-MTHF; ≥99.5 wt.%) and deuterated chloroform (≥99.8 atom % D), as well as the catalyst *p*-toluenesulfonic acid (PTSA; ≥98 wt.%), were purchased from Sigma-Aldrich (Sigma-Aldrich Ltd., St. Louis, MO, USA). *N*,*N*-dimethylformamide (DMF; >99.5 wt.%) was obtained from Carlo Herba (Carlo Erba Reagents S.A.S., Val De Reuil, France). Desmodur^®^ Eco N7300, a bio-based aliphatic 1,5-pentamethylene polyisocyanate, was obtained from Covestro (Covestro AG, Leverkusen, Germany). It has an NCO content of about 21.9%, a viscosity at 23 °C of ca. 9500 mPa.s, less than 0.3% of monomeric PDI and an equivalent weight (EW) of ca. 195. The 0.5 M and 0.1 M KOH solutions used in the determination of the polyols’ acid and hydroxyl values were purchased from Honeywell (Charlotte, NC, USA), and the solvent tetrahydrofuran (THF; 99.8 wt.%) was purchased from Fisher Scientific (Thermo Fisher Scientific Inc., Waltham, MA, USA). The DC01 carbon steel plates used were kindly provided by Voestalpine AG (Linz, Austria).

### 2.2. Thermochemical Liquefaction of Biomass into Polyol

Before the thermochemical liquefaction reaction, Stipa samples were dried in an oven at 60 °C, until no changes in weight were visible, and then ground in a Retsch©SM 2000 blade mill (Retsch GmbH, Haan, Germany) with a 4 mm sieve. Pinewood shaves were used as obtained.

Biomass, solvent, either 2EH or PG, (1:5 wt. of biomass to solvent), and PTSA catalyst (3 wt.%) were fed to a reactor and allowed to react at a moderate temperature of 160 °C for 90 min, with mechanical stirring in a nitrogen (N_2_) inert atmosphere. A Dean–Stark apparatus was used to capture moisture from the biomass and water formed during the reaction. After the set time, the reactor was allowed to cool down at room temperature, after which the contents were vacuum filtered. The solid biomass residue was separately washed with organic solvent and thoroughly dried at 60 °C until no meaningful weight change was observed, after which it was collected for further analysis. The process was adapted from other works from the group, described elsewhere [27,37,38]. The biomass conversion was calculated according to Equation (1):

(1) $$Biomass\;Conversion\;(\mathrm{wt}.\%)=\left(1-\frac{m_{residue}}{m_{residue}}\right)\times 100$$

where *m_residue_* is the mass (g) of solid biomass residues at the end of the reaction, and *m_biomass_* is the mass (g) of biomass fed to the reactor, on a dry basis.

The liquified biomass obtained from the reaction was subjected to a process of liquid–liquid extraction with water, in a proportion of 1:3 (*v*/*v*), and the liquified biomass organic fraction was retrieved. The organic fraction was further submitted to vacuum distillation to recover all the solvent from the mixture and obtain the biopolyol.

Using the above procedures, two distinct biopolyols, named PolyI and PolyII, were produced using 2EH as the solvent for thermochemical liquefaction, and pinewood and Stipa as biomasses, respectively. A third biopolyol, hereafter named PolyIII, was produced using pinewood biomass and a green solvent PG to replace the typically used 2EH solvent in the liquefaction process (Table 1).

### 2.3. BioPolyurethane (BioPU) Synthesis and Coating Preparation

BioPUs were prepared by a one-pot method, where the biopolyol is mixed with the bio-based aliphatic polyisocyanate Desmodur^®^ Eco N7300 (hereafter referred to as Eco N7300) at a 0.9 ratio of isocyanate (NCO) to hydroxyl (OH) value of polyol.

The polyol was dissolved in the green solvent 2-MTHF (50:50 wt.%), to allow for a good homogeneity of the reactants. The Eco N 7300 was added to the mixture, which was sonicated for 15 min at room temperature. Three distinct BioPU coatings with around 100 µm thickness, hereafter called BPUI, BPUII, and BPUIII, were manually deposited by the bar coating method on rectangular carbon steel substrates (surface area ~20 cm^2^) and allowed to dry at 50 °C over a period of at least 72 h. The substrates had been previously washed and sonicated in ethanol and acetone for 15 min each and dried with hot air.

### 2.4. Characterization Techniques

#### 2.4.1. Biopolyols’ Acid Value (AV) and Hydroxyl Value (OHV)

The acid value (AV) was determined by the method based on ASTM D 947 and DIN 51558 standards, and 1 g of polyol (titrand) dissolved in 50 mL of THF was titrated with a 0.1 N KOH solution (titrant) with a phenolphthalein indicator.

Hydroxyl value (OHV) was determined by titration method based on ASTM D 1957 and ASTM E222-10 standards. Approximately 1 g of polyol was dissolved in 50 mL of tetrahydrofuran (THF) and acetylated by 10 mL of 12.5 wt.% acetic anhydride solution in THF for 10 min under stirring. After this, 2 mL of water was added to react with the remaining excess of acetic anhydride for 30 min, stopping the acetylation process. The OH groups turned acetic acid, with a thymolphthalein indicator, were titrated using a 0.5 N KOH solution (titrant). A blank solution following the same procedure, without adding titrand, was prepared at the same time.

After the determination of the turning point of the titrations, the following formulas were applied:

(2) $$AV=\frac{{\left[C\right]}_{titrant}\times {MM}_{KOH}\times V_{KOH}}{m_{titrand}}$$

(3) $$OHV=\frac{{\left[C\right]}_{titrant}\times {MM}_{KOH}\times \left(V_{blank}-V_{KOH}\right)}{m_{titrand}}+AV$$

where AV is the Acid value (mg KOH g^−1^) and the OHV is the Hydroxyl value (mg KOH g^−1^). The *[C]_titrant_* is the concentration of titrant (N, normality) utilized and *MM_KOH_* is the molecular mass of KOH (56.1 g mol^−1^). The *V_blank_* is the quantity of titrant used in the titration of the blank solution (mL) and *V_KOH_* is the quantity of titrant used in the titration (mL). The *m_titrand_* is the mass of the titrated sample weighted (g).

#### 2.4.2. Physico-Chemical Characterization

The elemental composition of the biomass feedstock and respective polyols prepared were analyzed concerning their carbon (*C*), hydrogen (*H*), nitrogen (*N*), and sulfur (*S*) content, by an EMA 502 Elemental Micro Analyzer (VELP Scientifica Srl, Usmate Velate, Italy). The oxygen (*O*) content was estimated according to (Equation (4)):

(4) $$O\left(\%\right)=100-\left(C\left(\%\right)+H\left(\%\right)+N\left(\%\right)+S\left(\%\right)\right)$$

An Attenuated Total Reflectance-Fourier Transform Infrared Spectroscopy (ATR-FTIR) analysis of the polyols and BioPU were performed in a Spectrum Two from PerkinElmer equipped with a UATR accessory (PerkinElmer Inc., Waltham, MA, USA). The spectra were obtained using 32 scans of data accumulation at a resolution of 4 cm^−1^, in a wavenumber range of 4000 to 400 cm^−1^.

The thermal stability of the polyols and BioPU was analyzed by thermogravimetric analysis (TGA), using aluminium (Al) pans (ø 5.2 mm, h 2.5 mm), performed on a Hitachi-STA7200 (Hitachi High-Tech Corporation, Tokyo, Japan), from room temperature to 600 °C, at a heating rate of 10 °C min^−1^, under a 100 mL min^−1^ flow rate of nitrogen (N_2_; ≥99.99 wt.%) gas.

The ^1^H NMR spectra of the biopolyols were acquired in a Bruker AVANCE III RNM (Bruker Inc., Billerica, MA, USA), running at 400 MHz, with deuterated chloroform as solvent.

The hydrophobicity of the coatings was evaluated by measuring the optical contact angle (OCA) of water on dry BioPU samples through the sessile drop method. Static OCA measurements on the BioPU films were performed using a JAI CV-A50 (JAI Ltd., Copenhagen, Denmark) video camera attached to a Wild M3Z (Leica Microsystems, Wetzlar, Germany) microscope connected to a Data Translation DT3155 frame grabber and a micrometric syringe to dispense Millipore water droplets (2 to 3 μL) at room temperature. The acquisition and analysis of the images were performed using ADSA-P software (Axisymmetric Drop Shape Analysis Profile). A minimum of 10 measurements were taken in different regions of the coating surface.

Adhesion tests based on ASTM D4541 were performed on the BioPU coatings on carbon steel substrate (surface area ca. 20 cm^2^) by employing a PosiTest AT-A adhesion pull-off tester (DeFelsko Corporation, Ogdensburg, NY, USA). The tester measures the pulling force needed to detach a 20 mm diameter aluminium dolly adhered to the PU film, employing epoxy adhesive.

The Shore hardness of the biopolyurethanes was manually measured by X.F Shore A and D durometers, following the standard ASTM 2240. A minimum of 10 measurements were made, for about 3 s each, at room temperature.

Swelling tests were conducted on samples of BioPU blocks of ca. 100 mg in weight, in both solvents—Millipore water and DMF. The samples were weighed and submerged in individual containers with 20 mL of solvent at room temperature. The samples were taken out, dried on a paper towel, and weighed periodically over the course of 72 h. The swelling rate (*SR*) (Equation (5)) was calculated from the weight of the dried polymer ($w_{o}$) and swollen polymer ($w_{s}$).

(5) $$SR(\%)=\frac{w_{s}-w_{o}}{w_{0}}\times 100$$

The density of each polymer was estimated by the water displacement method at room temperature.

Dynamic mechanical analyses (DMA) were carried out on a DMA Q800 device (TA Instruments, New Castle, DE, USA). Multi-frequency strain of samples (20 × 14 × 3 mm) was performed with a heating rate of 2 °C·min^−1^ from 30 to 150 °C, operating at a frequency of 1 Hz and an amplitude of 15 µm. The cross-linking density (*ν_x_*) of all the BioPUs was estimated according to the kinetic theory of rubber elasticity (Equation (6)) [57]. The molecular weight of the chain between cross-linking points (*M_x_*), inversely proportional to $v_{x}$, was calculated according to Equation (7):

(6) $$v_{x}\left(\mathrm{mol}m^{-3}\right)=\frac{{G^{\prime }}_{T^{\prime }}}{3RT^{\prime }}$$

(7) $$M_{x}\left(\mathrm{Kg}{\mathrm{mol}}^{-1}\right)=\frac{\rho_{}}{3v_{x}}$$

where *T*′ is the temperature of samples in the rubbery plateau region at Tg + 50 °C, and *G*′_*T*′_ is the corresponding storage modulus at *T*′. *R* is the ideal gas constant (8.314 m^3^ Pa K^−1^ mol^−1^).

#### 2.4.3. Electrochemical Impedance Spectroscopy (EIS)

Electrochemical impedance spectroscopy (EIS) was used to study the barrier properties of the coating applied on steel immersed in NaCl 0.05 M. A Gamry potentiostat Reference REF 600 (Gamry Instruments, Pennsylvania, USA) apparatus was used for the measurements, carried out on a conventional three-electrode cell configuration inside a Faraday cage. A saturated calomel electrode (SCE; model HI5412, Hanna Instruments, Woonsocket, RI, USA) was used as the reference electrode, a platinum coil as the counter electrode, and the coated DC01 carbon steel, with a total exposed area of around 3.46 cm^2^, was used as the working electrode. The EIS measurements were carried out at open circuit potential under a 10 mV (r.m.s.) sinusoidal perturbation and in the frequency range from 50 kHz to 5 mHz. ZView^®^ software (ScribnerAssociates, Southern Pines, NC, USA) was used to treat the obtained EIS data.

## 3. Results and Discussion

### 3.1. Thermochemical Liquefaction of Biomass

The objective was to obtain green polyols from the thermochemical liquefaction of biomass to directly replace petroleum-based polyols in the formulation of PUs. The process parameters used in the present work were based on optimization studies, recently performed by the team, which can be found in the literature for the liquefaction of pinewood biomass [27,34]. Table 2 summarizes the results of the thermochemical liquefaction of the two feedstocks, pinewood and Stipa, under the same conditions (temperature, time, and catalyst amount). Overall, the process resulted in good conversion rates for both biomasses, with the results for pinewood being 77% and 79% using solvents 2EH and PG, respectively, which are similar values to those found in the literature [27,34]. Although no optimization was developed, at the end of the process, around 70 to 75 wt.% of the solvent utilized in the synthesis was recovered by low-pressure evaporation of the liquified biomass. For the Stipa biomass, to the best of our knowledge, no recent publications have mentioned the thermochemical liquefaction of this feedstock. The conversion rate obtained from the thermochemical liquefaction of Stipa biomass is greater than that obtained for the fast pyrolysis (FP) process, where the conversion was reported to be 57% at 500 °C with a heating rate of 150 °C min^−1^ [58], and comparable with the rates obtained for pinewood biomass. The result demonstrates that, although the process can still be optimized for the specific feedstock, it is possible to obtain good conversion rates for widely different feedstocks.

### 3.2. Polyol Acid Value (AV) and Hydroxyl Value (OHV)

The polyols’ AV and OHV are of significant importance since PUs are prepared from a reaction between a polyisocyanate and a polyol at a specific free NCO/OH ratio. These values were determined by titration. An accurate OHV depends on AV, which is one parameter in equation III, and results from the acidic lignocellulosic material, namely from the oxidation of carbohydrates and lignin during liquefaction. The AV of all polyols developed in this work was below 20 mg KOH g^−1^, and their OHVs varied between 110 and 160 mg KOH g^−1^ (Table 3), which should be due to the different feedstocks and solvents used. Although the OHV can vary widely, from as low as 46 mg KOH g^−1^ to as high as 1270 mg KOH g^−1^, the obtained values are still in line with those reported in the literature for bio-oil polyol fractions, which usually vary from 150 to 350 mg KOH g^−1^ [37,38,45]. For the AV, reported values are between 0 and 40 mg KOH g^−1^. The OHV of polyol extracts tends to be lower than that of the liquified product without liquid–liquid extraction pretreatment [45,59]. Nonetheless, we concluded that the polyols obtained possess suitable OHV values for the preparation of PUs.

### 3.3. Elemental Analysis

Table 4 shows the reduction of the O/C ratio in the polyols produced by thermochemical liquefaction, which lies between 0.34 and 0.41, compared to that of the biomass feedstock, which generally is ca. 1 [27,31,33,34,60]. The reduction of the oxygen to carbon ratio, when compared to that from fresh biomass, can occur during the depolymerization of the biomass’ lignocellulosic polymers. Reactions such as hydrolysis, hydrogenolysis, dehydration, and hydrodeoxygenation occur during the thermochemical liquefaction process, possibly producing water and light single-carbon molecules, such as CO or CO_2_, as subproducts, which are removed during the process [29,35,61]. The formation of these small molecules can be due to the cracking of the ether bonds (C-O-C) of the cellulose structure [61]. Therefore, the O/C ratio can be seen as a measure of the depolymerization of the resulting biomass subproduct. The presence of the S element in PolyI and PolyII is due to the PTSA catalyst used during the synthesis process. The elements N and S are present in the polyols in very small amounts (≤1.5%) and are not considered significant.

### 3.4. Nuclear Magnetic Resonance (NMR) Spectroscopy Analysis

**Figure 3:**
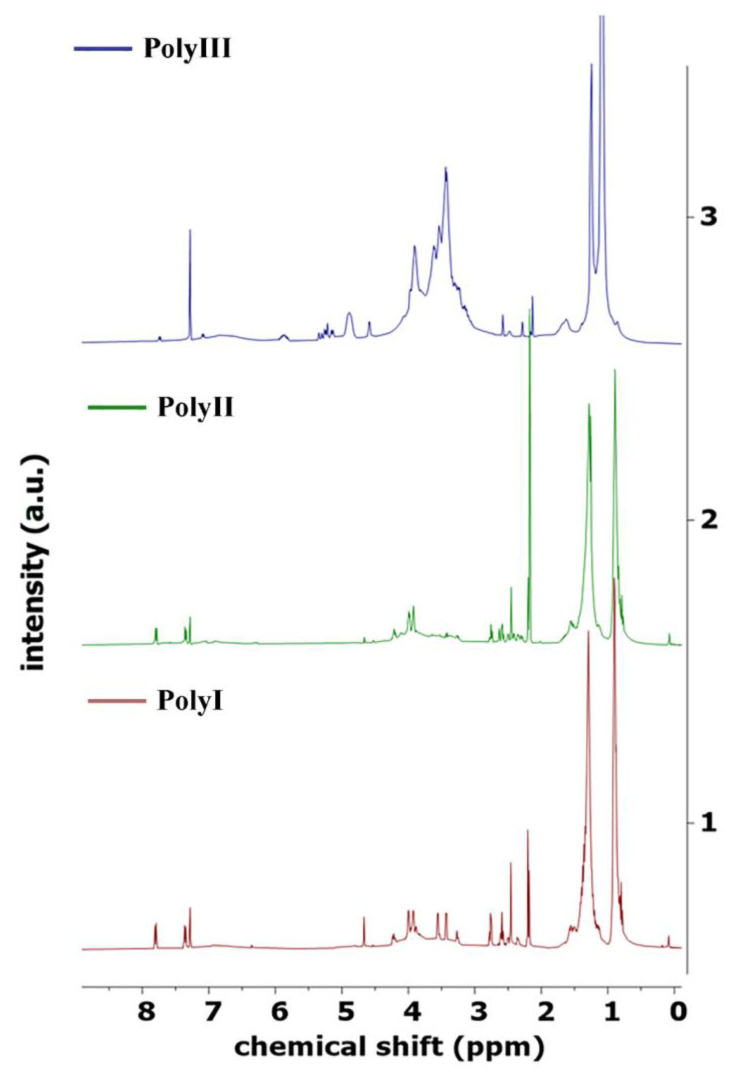
^1^H NMR spectra of the biopolyols PolyI, PolyII, and PolyIII, run at 400 MHz, with deuterated chloroform as solvent.

NMR spectroscopy is a powerful technique that can be used to obtain approximate ratios of chemical environments of protons and carbon atoms in bio-oils. An in-depth analysis of bio-oils using NMR may allow for clearer characterization, including relative substructure compositions such as the presence of functional groups, degrees of saturation, branching, or aromatic content [62,63,64,65,66,67]. The ^1^H of the three different biopolyols—PolyI, PolyII, and PolyIII—is shown in Figure 3. The integral values of the selected regions of the spectra are shown on a percentage basis in Table 5.

Regarding the proton NMR spectra of the three samples, the similarity between PolyI and PolyII is clear, which were produced from different feedstocks but utilizing the same conditions, including the same solvent—2EH. The most upfield region, between 0.5 and 1.5 ppm, is assigned to aliphatic protons attached to carbon atoms. For PolyI and PolyII, this region was by far the most populated, with values between 56 and 63%. The result reveals the prevalence of aliphatic hydrocarbon chains of two or more bonds, which are more hydrophilic in nature. The region at 1.5–3.0 ppm pertains to protons bonded to a C=C double bond of aromatics, olefins, or protons that are two bonds away from N- or O-heterocyclic compounds. In this region, the percentage values between 9.0 and 15.2% show the low degree of unsaturation of the compounds present in the polyol. At 3.0–4.4 ppm, the proton peaks are assigned to aliphatic alcohol, ether, or possibly to methylene groups bonded to two aromatic rings (a possible derivate from lignin’s partial decomposition) [62]. For PolyI and PolyII, around 17% of the protons are in this region, being the second-most-populous region after the aliphatic upfield region. For PolyIII, the region is much more populated, accounting for 43.1% of all the protons. The following section of the ^1^H spectra, 4.4–6.0 ppm, is attributed to lignin-derived methoxyphenols and carbohydrates and contains only about 2.8 to 6.6% of the total protons, being the least-populated region of the spectra. It is said that these protons are more prevalent in herbaceous grasses [62]. However, that is not very evident in PolyII, which uses the Stipa grass as feedstock. This result may be due to the fibrous nature of the latter. Still, it may also be due to the liquid–liquid extraction pretreatment, which extracts some of these molecules from the polyol derived from the cellulose and hemicellulose degradation. The aromatic region of the spectra, from 6.0 to 8.5 ppm, is assigned to protons in benzenoids and N- or O- heteroaromatic compounds. This region contains between 4 and 8% of all protons in the polyols. Overall, the polyols PolyI and PolyII are shown to contain elevated quantities of protons bonded to aliphatic and alcohol moieties, ascribed to the region between 0.5 and 4.4 ppm. The abundance of these structures may be partially related to the solvent 2EH used during the thermochemical liquefaction reaction. In contrast, PolyIII is shown to have fewer aliphatic protons in the range of 0.5 to 3.0 ppm and presents more alcohol-bonded protons, ca. 43%, instead. Again, this result may be the influence of the solvent PG utilized during the liquefaction of the biomass, as the PG is a diol and possesses a shorter chain than 2EH. The reactions in an organic medium strongly depend on the solvent used. As a rule of thumb, the solvent should have strong interactions with the biomass macromolecules and should be a product of the reaction itself. As liquefaction consists of a series of de-polymerizations, alcohol solvents can react with the degradation products of the macromolecules and be integrated into the structure of the resulting polyol mixture [30]. One example of such possible reactions is the esterification of carboxylic acid compounds with the solvent PG [30,39,40].

### 3.5. Attenuated Total Reflection-Fourier Transform Infrared Spectroscopy (ATR-FTIR) Analysis

**Figure 4:**
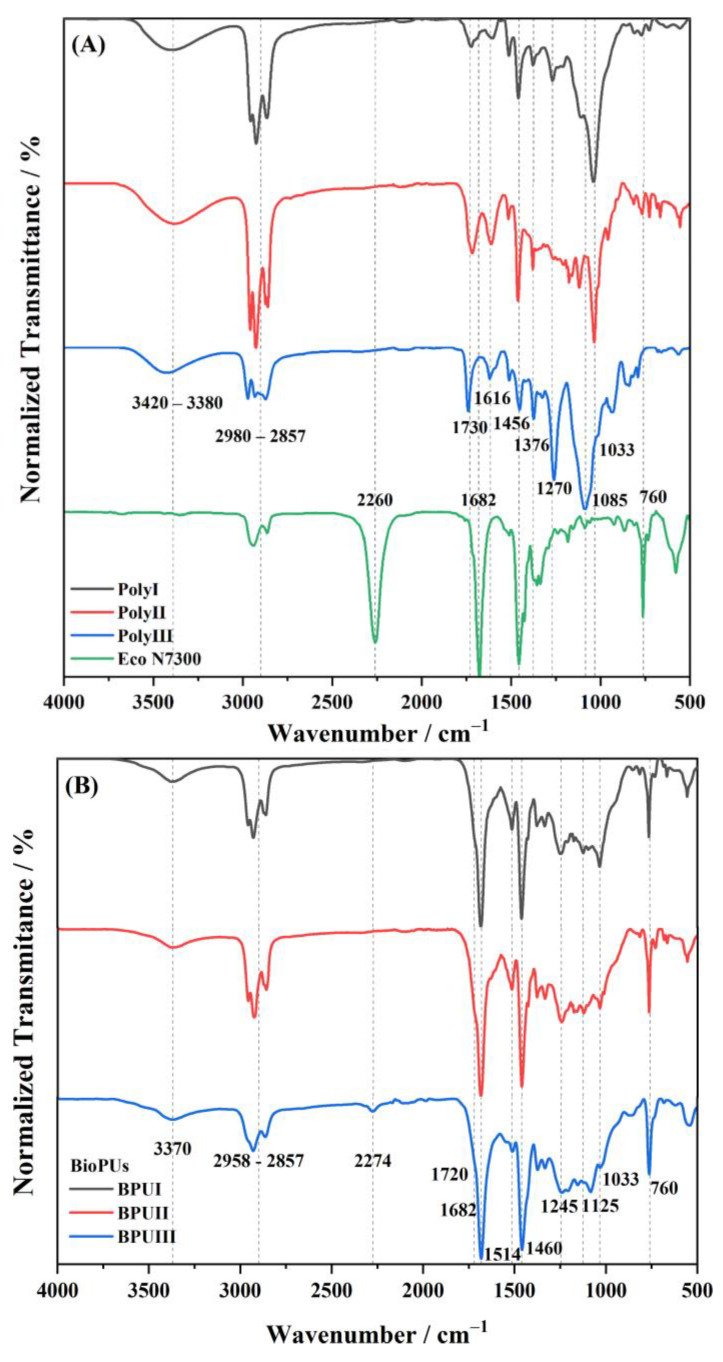
ATR-FTIR spectra of (**A**) Desmodur^®^ Eco N7300 and the polyols obtained from the thermochemical liquefaction as well as (**B**) respective BioPU coatings.

The ATR-FTIR spectra of the polyols obtained from the thermochemical liquefaction of pinewood and Stipa biomass with 2EH and PG solvents are presented in Figure 4A and the spectra of the respective BioPU are presented in Figure 4B. The functional groups ascribed to the most prominent peaks are listed in Table 6.

The polyols spectra in Figure 4A present, as expected, peaks related to the most characteristic biopolyols functional groups. A small band at 3420–3380 cm^−1^, attributed to hydroxyl groups, reveals the presence of alcohols and carboxylic acids [27,34,60]. The characteristic peaks between 2980 and 2857 cm^−1^ show the presence of CH_2_, and CH_3_ aliphatic chains [12,27,34,68,69]. In this region of the spectra, PolyIII shows considerably smaller peaks, which may indicate fewer aliphatic chains, and this is corroborated by NMR analysis of the biopolyol. A weak peak assigned to C=O stretching, around 1738–1716 cm^−1^, relative to hemicellulose and lignin derivatives is visible [12,27,68,69]. Levulinic acid, which results from the solvolysis of cellulose, is also known to display a strong peak at 1720 cm^−1^ [70]. The peak at 1459–1451 cm^−1^ is relative to C–H bending in alkyl groups and alkanes, complementing the peaks at 2980–2857 cm^−1^ [34,68,69]. PolyIII (Figure 4A) shows a large peak at 1264 cm^−1^, associated with C-O stretching in aromatic esters or alkyl aryl ethers. All polyol spectra show large peaks between 1085 and 1034 cm^−1^, which are usually assigned to vibrations of lignin derivatives, and also cellulose- and hemicellulose-derived oligomers, which are still present in the polyol sample [12,34]. For PolyIII, the larger band at 1085 cm^−1^ may indicate the presence of more ester compounds, which can be from parallel esterification reactions [39,40] of PG solvent with carboxylic acid products of the depolymerization. This would be in agreement with the previous peak, at 1264 cm^−1^.

Relative to the spectra of the BioPUs (Figure 4B), the peaks at 3370 cm^−1^ (N–H stretching) [12,15,71], 1720 cm^−1^, and 1680 cm^−1^ (C=O stretching), coupled with the very reduced or even absent peak at 2274 cm^−1^ (NCO) [12,15] and reduced OH stretching band, prove the formation of urethane groups, by the reaction of NCO and OH groups and, therefore, the production of the BioPU material. The peak at 1514 cm^−1^ is commonly attributed to N–H deformation and C–N stretching vibrations. The peaks at 1125 cm^−1^ and 1033 cm^−1^ are related to C–O–C vibrations in PUs but can also be attributed to lignin derivatives from the polyol. The band between 1780 and 1600 cm^−1^ is particularly difficult to interpret as it encompasses several individual absorption bands, either from the raw materials or from the BioPU material. This band is mainly composed of the carbonyl vibration of urethane and urea linkages, including the isocyanurate ring structure of Eco N7300. The latter also contributes to the peaks at 1514 and 760 cm^−1^. Overall, the spectra of the BioPUs are as expected and in accordance with the literature.

Noteworthily, the BPUIII spectrum shows a small peak relative to free NCO at 2274 cm^−1^, indicating that not all the free isocyanate reacted with the hydroxyl groups. This may indicate some limited reactive capacity from part of the hydroxyl groups towards the isocyanate and can also be related to restrictions in the mobility of NCO functional groups with the increase of cross-linking density.

**Table 6:** ATR-FTIR band assignment for the most prominent peaks of the polyols and BioPU spectra.

Wavenumber/cm^−1^		Functional Group	Assignment	Source
Polyols	BioPU			
3420–3380	–––	–OH stretching vibration	Alcohols, carboxylic acids	[27,34,60]
–––	3370	–N–H stretching vibration	Urethane groups	[12,15,71]
2980–2857	2958–2857	C-H asym./sym. stretching vibration (–CH_2_, –CH_3_)	Methyl and methylene groups	[12,27,34,68,69]
–––	2274–2260	–NCO vibration	Isocyanates	[12,15]
1738–1716	1720	–C=O stretching vibration	Ketones, esters, carboxylic acids, urethane groups	[12,27,68,69,70]
–––	1682	–C=O stretching vibration	Urea groups, isocyanurates	[68,69]
1616	1620	–C=C– stretching vibration	Aromatic rings	[27,34]
1516–1509	1515–1514	–N–H deformation, –C–N– stretching vibration	Amine groups, urethane (amide) groups	[72,73,74,75]
1459–1451	1462–1460	–C–H bending vibration	Alkyl groups, alkanes	[34,68,69]
1379–1372	1380–1378	Aromatic –C–H deformation	Syringyl rings	[27,34]
1272–1264	1249–1243	–C–O vibration; aromatic ring vibration	Guaiacyl rings	[12,34]
1085–1034	1125; 1033	–C–O–, –C=C–, –C–O–C– stretching vibration	Cellulose, hemicellulose, lignin	[27,34,69]
934	–––	–C=C– bending vibration, –C–H deformation	Alkenes, glycosidic linkages	[16,31,76,77]
848	–––	–C=C– bending vibration, –C–H vibration	Alkenes, aromatic rings	[27,60]
–––	760	–C–N vibration	Isocyanurates	[69,78]

### 3.6. Thermogravimetric Analysis (TGA)

**Figure 5:**
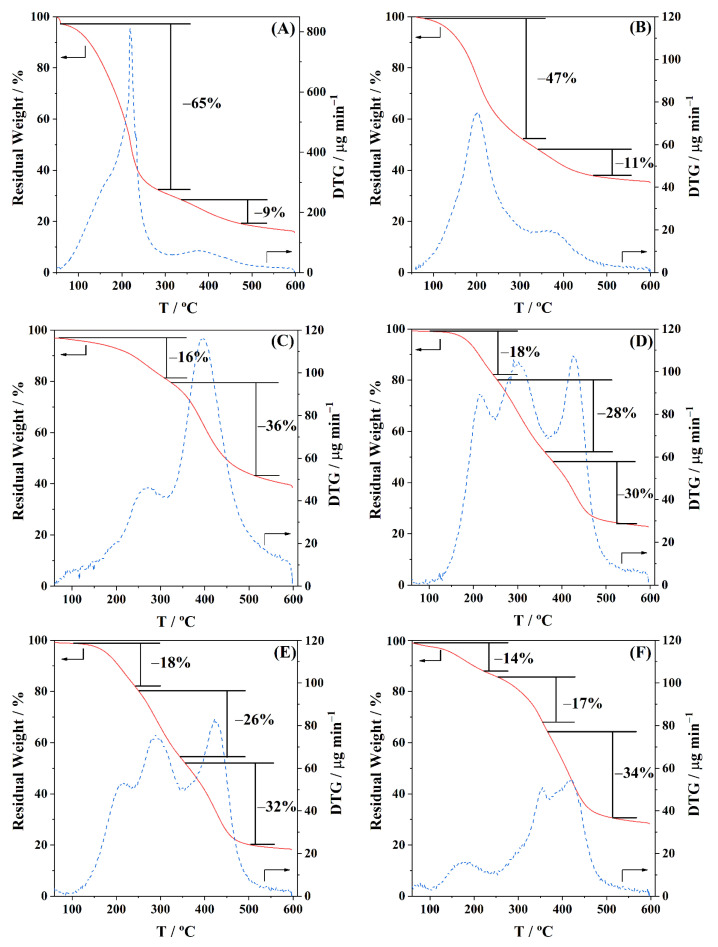
TGA (solid lines, axis on the left), and DTG (dashed lines, axis on the right) of the biopolyols, (**A**) PolyI, (**B**) PolyII, and (**C**) PolyIII, as well as the respective BioPU, (**D**) BPUI, (**E**) BPUII, and (**F**) BPUIII, under N_2_ atmosphere, up to 600 °C.

The TGA results of the three distinct polyols produced, as well as the respective BioPU coatings, are shown in Figure 5. The values of temperature and weight loss at each step as well as the final residual weight at 600 °C are listed in Table 7.

Both PolyI and PolyII (Figure 5A,B, respectively) show relatively similar behaviour, with onset degradation temperatures of ca. 100 °C and DTG peaks between 200 and 250 °C, probably corresponding with the degradation or volatilization of the polyol lighter components. The mass loss in this first degradation step was 65% and 42% for PolyI and PolyII, respectively. A second degradation step, between 300 and 450 °C, shows a mass loss of 9 to 11%, most likely resulting in the formation of ash and carbon [34], non-degradable up to the upper temperature of analysis. PolyIII (Figure 5C) shows a first step starting only at above 150 °C and reaching 300 °C, losing only ca. 16% of the mass. The PG solvent still present in the polyol, as corroborated by the RMN analysis, may contribute to this degradation step since it is eliminated only above 190 °C. The second degradation step is much more prominent, with 32% of the mass being lost between 300 °C and 450 °C, possibly due to the presence of lignin derivatives with higher molecular weights. Overall, the TGA results agree with the previous literature [34]. Overall, both polyols, PolyI and PolyII, exhibit similar shapes of the thermograms, revealing a low variability of composition, despite the differences in the initial feedstock. On the other hand, the nature of the solvent employed plays a more important role in the thermal behaviour of the obtained polyols (PolyI and PolyIII).

Regarding the thermal decomposition of the BioPU coatings (Figure 5D–F, for BPUI, BPUII, and BPUIII, respectively), three main steps of degradation are observed [19,79,80,81,82]. The weight lost in each step and the temperature of the corresponding DTG peak, as well as the final char residue, are presented in Table 7. In the beginning, below 100 °C, some residual weight is lost, most possibly related to some 2MTHF solvent or moisture, still trapped in the polymeric matrix. The first degradation step is usually assigned to the decomposition of active hydrogen sources or the degradation of the PU soft segments. The second and third degradation steps are attributed to the degradation of hard segments of the PU matrix, and also to the degradation of cellulose and lignin derivatives. When comparing the different BioPU coatings prepared, BPUI and BPUII (Figure 5D,E) show similar behaviour and good thermal stability below 100 °C; however, BPUIII (Figure 5F) is the one that shows the highest thermal resistance above 200 °C and a higher amount of char residue at 600 °C, 28 wt.%, which is in accordance with the result of the respective polyol, PolyIII.

The results obtained are comparable with the literature reporting that BioPU presents an onset of degradation just above 100 °C, revealing adequate thermal stability for applications subjected to mild temperatures.

### 3.7. Optical Contact Angle (OCA)

**Figure 6:**
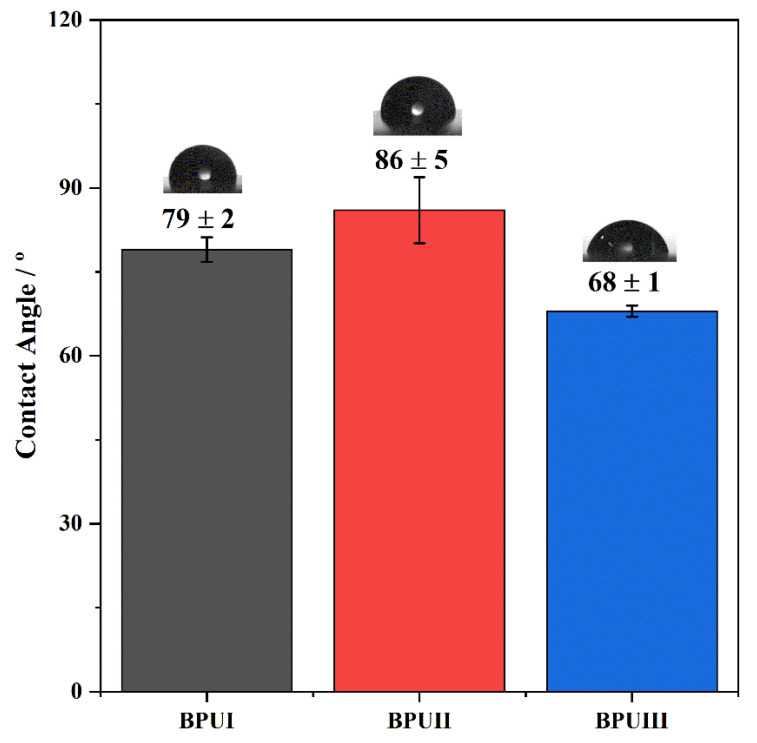
The static optical contact angle of water in BPUI, BPUII, and BPUIII sample coatings.

The static contact angle of water on the BioPU coatings was measured for BPUI, BPUII, and BPUIII (Figure 6). The contact angle is illustrative of the hydrophobic nature of polymeric coatings, which can be relevant for the material’s performance when exposed to natural weathering, especially regarding corrosion protection. All coatings present mild hydrophobicity, between 68° and 86°, with BPUII presenting the highest contact angle of water. It is known that the hydrophobicity of bio-based PU decreases with the presence of lignin derivatives, possibly due to an increase of free hydroxyl groups [83], which might explain the mild hydrophobicity obtained with these biopolyols. For BPUIII, a bigger number of unreacted, free hydroxyl groups and the abundance of alcohol functional groups to the detriment of more aliphatic structures, as is visible in the NMR and FTIR analysis, may explain its characteristic low hydrophobicity revealed by the contact angle of 68°. Comparing PolyI and PolyII, again they present similar results, with PolyII exhibiting a slightly higher static contact angle of water—86°. This can possibly be due to a higher quantity of aromatic structures, as the NMR analysis reveals. These values are in line with the literature for unmodified bio-based PUs [19,23,24,79].

### 3.8. Hardness, Swelling, and Adhesion Tests

The hardness of the synthesized BPU polymers was evaluated by an X.F Shore D durometer, where each cured polymer block was subjected to an average of 10 measurements in different locations, at room temperature (Table 8).

All BPUs synthesized are considered hard materials in the Shore Hardness Scale, presenting average values between 46 and 65 Sh D, with BPUIII showing the highest value of hardness. This result is in accordance with the previous results of TGA, where it was visible that BPUIII had the highest thermal resistance. The NMR analysis also shows that BPUII had a higher prevalence of aromatic compounds compared with BPUI, which might explain the higher hardness. These values of hardness are in line with other reported values for hard polyurethane polymers, which are above 32 Sh D [68,84].

Swelling tests were performed for all BioPU samples in water, for a period of 72 h, at room temperature. 

Table 8 shows the results of the measurements, and as expected the BioPU coatings have little affinity with water, only showing a *SR* of 4 to 6% when submerged for 72 h.

**Figure 7:**
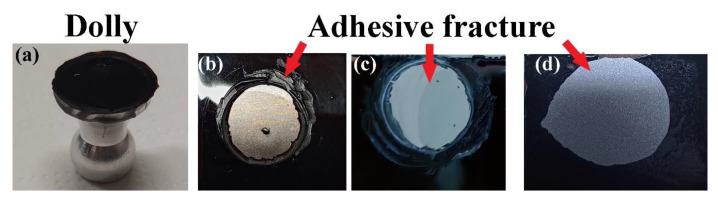
Pictures of (**a**) dolly used in the pull-off adhesion tests of coating samples (**b**) BPUI, (**c**) BPUII, and (**d**) BPUIII.

The adhesive strength of the BioPU coatings on carbon steel substrates was assessed by performing pull-off adhesive tests (Figure 7). The coatings show averaged adhesive forces between 0.84 and 2.18 MPa (Table 8), with BPUI and BPUII showing the best results.

Overall, the tests show near to 100% adhesive fracture, with interfacial bond failure between the coating and the substrate, which is aligned with the results found in the literature for bio-based PU systems on steel, as well as on other metal substrates [19,83,85]. It is speculated that the relatively high adhesion pull-off strength of the bio-based PU coatings may be related to free, unreacted OH groups, in this case present in the polyol phenolic segments of the BioPU, allowing for strong hydrogen bonding interactions with the metal substrate [19,85]. Additionally, there is the possibility for the formation of covalent bonds between free isocyanate functional groups and hydroxides present on the metal surface, leading to the formation of a urea-like bond between the PU and the substrate [86].

Analyzing the results obtained for the BPUIII coating, the low adhesive strength is most possibly linked with the molecular structures of PolyIII, the polyol used in its synthesis. The TGA showed PolyIII to possibly have some alkylphenol derivatives with higher molecular weight, and NMR analysis has shown PolyIII to have a significant signal related to alcohols. The presence of more alcohols and fewer aliphatic structures, as seen in the NMR spectra, can make BPUIII structures harder, more brittle, and prone to fracture.

The pull-off adhesion strength of these coatings is in line with the reported values in the literature [87,88,89]. For instance, Mayer et al. [87] reported values up to 3.12 MPa, but the substrate of the study was aluminium, and this larger value was obtained for a modified PU coating on degreased and abrasive blasted substrate. Griffini et al. [89] achieved 0.9 MPa for a lignin-derived PU coating on stainless steel, and 1.5 MPa for the same coating on aluminium substrates. De Haro et al. [19] presented adhesion strength values between 1.28 and 1.4 MPa for lignin-based PU coatings on a stainless-steel substrate. Additionally, it is important to mention that, except for the cleaning with acetone through sonication, no other physicochemical treatments, such as acid pickling or sandblasting, were performed on the carbon steel substrates before the coating deposition.

### 3.9. Dynamic Mechanical Analysis (DMA)

**Figure 8:**
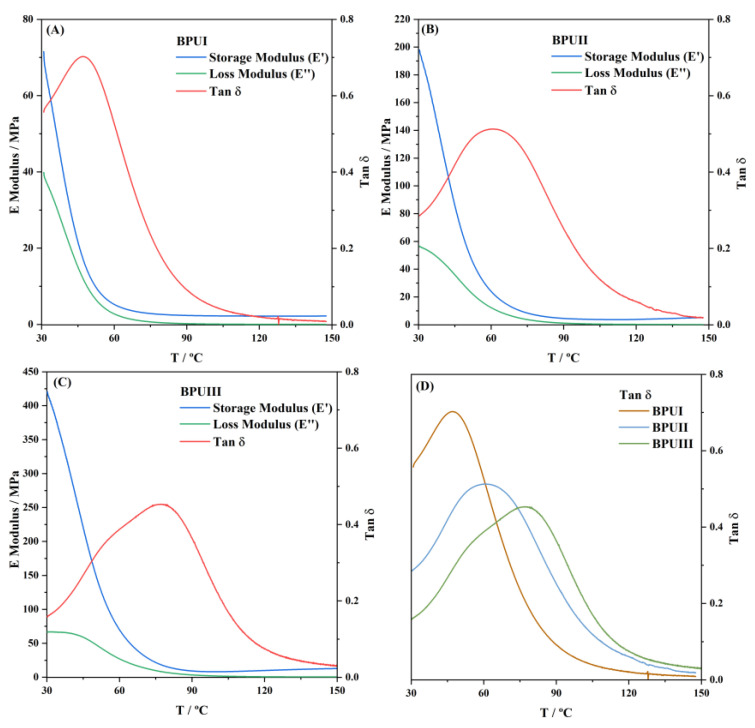
DMA of the BioPU, (**A**) BPUI, (**B**) BPUII, and (**C**) BPUIII, from 30 to 150 °C, with a heating rate of 2 °C min^−1^, operating at 1 Hz, as well as (**D**) comparison of the tan δ for the three BioPU.

The three synthesized polymers were analyzed by DMA (Figure 8). The storage modulus (G′), which reflects the solid elastic behaviour; the loss modulus (G″), which expresses the viscous liquid behaviour; and the loss factor (tan δ), a measure of the damping capacity, were recorded from 30 to 150 °C. The glass transition temperature (Tg) was determined as the maximum loss factor. The tan δ, calculated as a ratio of energy dissipated to energy accumulated in a process, is below 1 for all the three polymers, with G’ being dominant over G″, revealing the characteristic solid elastic behaviour of these materials. The results show that BPUI exhibits lower Tg and a higher G″, reflecting a stronger viscous liquid component and, therefore, higher flexibility than BPUII and BPUIII. As mentioned previously, the PG solvent employed in the biopolyol utilized as raw material for BPUIII might be responsible for the high Tg (77 °C), generating a coating too stiff for this application. The Stipa-based biopolyol leads to a BioPU with higher stiffness compared with pinewood-based biopolyol (larger Tg, 61 °C versus 47 °C), possibly due to the presence of a higher number of aromatic compounds, as seen by the NMR analysis. The cross-linking density (*ν_x_*) and molecular weight of the chain between cross-linking points (*M_x_*) were estimated by the kinetic theory of rubber elasticity (Table 9). These results are in line with some of the previous results obtained, which show that BPUIII might have an excess of cross-linking, making it harder and with higher thermal resistance, but also stiffer and, therefore, more brittle, revealing lower adhesion to carbon steel. Overall, the values of Tg obtained, although on the higher side, are still in line with PU materials reported in the literature [57].

### 3.10. Electrochemical Impedance Spectroscopy (EIS)

**Figure 9:**
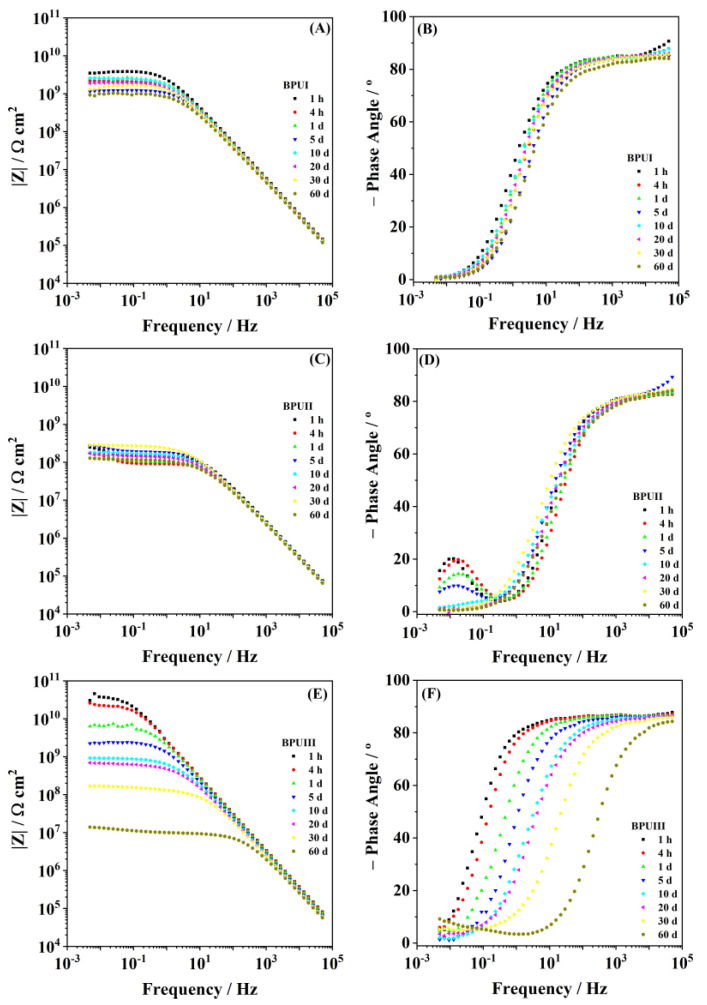
Bode plots for the BioPU samples, (**A**,**B**) BPUI, (**C**,**D**) BPUII, and (**E**,**F**) BPUII, for a period of 60 days immersed in 0.05 M NaCl solution.

The EIS Bode plots for the BioPUs applied on steel are presented in Figure 9. In the high-frequency region, the plots show a broad time constant with phase angle values below −80°, and a stable capacitive slope, which evidences the coatings’ good barrier properties. The value of the impedance modulus in the low-frequency region is regarded as a semi-quantitative indication of the coating protectiveness. The higher the low-frequency impedance modulus is, the higher is the anti-corrosion performance. The results show (Figure 9A–F) that all coatings display |Z|_0.005 Hz_ above the reference values (1 × 10^7^ Ω cm^2^) during the first 60 days of immersion, showing values of 9.3 × 10^8^, 1.3 × 10^8^, and 1.4 × 10^7^ Ω cm^2^, for BPUI, BPUII and BPUII, respectively. In the Bode plots of BPUIII (Figure 9E,F), the shift of the breakpoint frequency to higher values is well evidenced. This parameter, defined as the frequency of the 45° phase angle, is correlated with the relative increase of electrochemically active surface area, and its shift to higher values over time indicates the gradual deterioration of the barrier properties and the onset of corrosion at the coating-steel interface, which is noted by the onset of a new time constant in the low-frequency region [90]. The onset of corrosion is also noticed for the BPUII coating despite the relatively stable low-frequency impedance values (Figure 9C,D).

**Figure 10:**
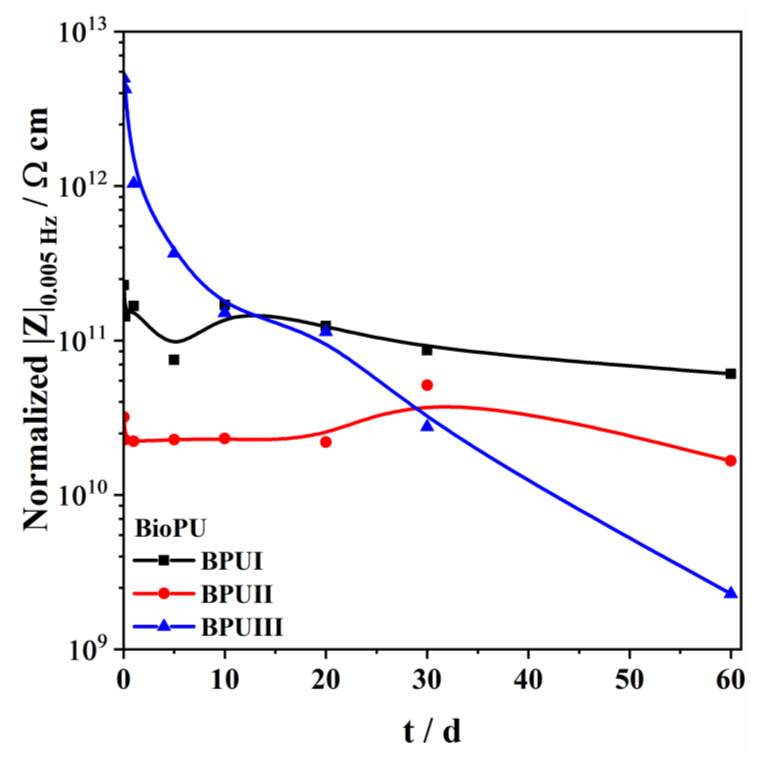
Evolution of the impedance module in the low-frequency region, |Z|_0.005Hz_, normalized for the coating thickness, for a period of 60 days immersed in 0.05 M NaCl solution.

As the coating thickness influences the coating protective efficacy, a quantitative comparison between the performances of different BioPUs was obtained by normalizing the |Z|_0.005 Hz_ values over the coating thickness (Figure 10). BPUI presents stable values, having a normalized impedance module |Z|_0.005 Hz_ of 6.1 × 10^10^ Ω cm at day 60, while BPUII presents a value of 1.7 × 10^10^ Ω cm at the same period. Despite BPUIII showing the highest normalized impedance module |Z|_0.005 Hz_, of 4.2 × 10^13^ Ω cm in the first few hours, the impedance value steadily falls over the period of 60 days, ending at 2.3 × 10^9^ Ω cm.

According to the literature, reports on green PU coatings usually present normalized values of the impedance module in the low-frequency region, with |Z|_0,_ between 1 × 10^10^ and 1 × 10^11^ Ω cm [8,12,15,90,91], which is consistent with the obtained results. Analyzing the results, it is concluded that BPUI presents the best barrier properties and the most effective anti-corrosion properties as no time constant could be noticed in the low-frequency region during the immersion period. Comparing the findings for BPUI and PBUII coatings, it can be suggested that variations in the feedstock used for the synthesis of the polyols affect the BioPU coating protection capacity since the presence of a low-frequency time constant in BPUII evidences activity at the steel interface. The solvent employed in the liquefaction process also appears to play a role in the BioPU coating protective performance.

## 4. Conclusions

Three bio-based polyols were prepared from the thermochemical liquefaction of two biomass feedstocks, namely pinewood and *Stipa tenacissima*. The thermochemical liquefaction process resulted in a conversion from biomass to biopolyol from 72 to 79 wt.%, depending on the biomass feedstock and solvents employed. Pinewood biomass and propylene glycol solvent resulted in larger conversion rates to biopolyol (PolyIII), and larger OHV (160 mg KOH g^−1^). The polyols were also characterized by elemental analysis (EA), NMR analysis, ATR-FTIR analysis, and TGA, revealing the presence of phenolic and aliphatic compounds with hydroxyl (OH) functional groups. Biopolyols obtained when 2-ethyl hexanol was employed as the solvent (PolyI and PolyII) have similar chemical structures, despite the difference in the initial feedstock. According to the TGA results, the biopolyol from pinewood and propylene glycol, PolyIII, may possess lignin derivatives with higher molecular weight, or more cross-linked structures, since it exhibits higher thermal resistance and char residue at 600 °C. The polyols obtained were considered suitable for the preparation of BioPU coatings. Hydrophobic and good quality coatings were produced from the reaction of each of the developed polyols with a bio-based polyisocyanate (Desmodur^®^ Eco N7300), in an NCO/OH ratio of 0.9, and manually deposited by bar coating on carbon steel substrates. The formation of urethane moieties and the complete or extensive reaction of isocyanate species was confirmed by ATR-FTIR spectroscopy. Noteworthily, the coating prepared with PolyIII (BPUIII) still reveals the presence of free isocyanate (NCO) species, which may indicate some limited reactivity between the OH and NCO species. This coating also exhibits lower adhesive strength, in line with a higher cross-linking density and hardness. On the other hand, BioPU coatings derived from PolyI and PolyII (BioPUI and BioPUII) are the ones with higher adhesive strength, up to 2.2 MPa, indicating relatively good compatibility with the carbon steel substrate. Regarding corrosion resistance, assessed by electrochemical impedance spectroscopy (EIS), all coatings display an impedance modulus in the low-frequency region—|Z|_0.005 Hz_, above 1 × 10^7^ Ω cm^2^—with BPUI, BPUII, and BPUIII presenting values in the order of 9.3 × 10^8^, 1.3 × 10^8^, and 1.4 × 10^7^ Ω cm^2^, respectively, after a period of 60 days in 0.05 M NaCl solution. The most effective coating is the BPUI since no signs of corrosion onset could be observed in the phase angle plot.

## Figures and Tables

**Table 1:** Components used for polyols production and corresponding polyols abbreviations.

Biopolyol Acronym	Biomass	Solvent
PolyI	Pinewood	2-Ethylhexanol
PolyII	Stipa	2-Ethylhexanol
PolyIII	Pinewood	Propylene glycol

**Table 2:** Conversion rates of thermochemical liquefaction of pinewood and Stipa biomass with solvents 2EH and PG.

Biomass	Solvent	Conversion/wt.%
Pinewood	2EH	76.7 ± 4.7
Stipa	2EH	71.9 ± 3.3
Pinewood	PG	79.3 ± 2.4

**Table 3:** Acid Value (AV) and Hydroxyl Value (OHV) of the polyols obtained from pinewood and Stipa biomass thermochemical liquefaction PolyI, PolyII, and PolyIII.

Polyol	AV/mg KOH g^−1^	OHV/mg KOH g^−1^
PolyI	8 ± 4	110 ± 11
PolyII	16 ± 5	160 ± 23
PolyIII	11 ± 4	139 ± 14

**Table 4:** Elemental analysis results of the pinewood and Stipa biomass feedstocks and the produced polyols, PolyI, PolyII, and PolyIII.

Sample		Element/wt.%					O/C Ratio
		C	O	H	N	S	
**Biomass**	Pinewood	48.08 ± 0.07	44.78 ± 0.52	5.92 ± 0.08	1.24 ± 0.36	0.00	0.93
	Stipa	46.48 ± 0.07	44.93 ± 0.52	6.42 ±0.08	2.18 ± 0.36	0.00	0.97
**Polyols**	PolyI	67.28 ± 0.67	23.41 ± 0.77	8.37 ± 0.05	0.37 ± 0.20	0.57 ± 0.06	0.35
	PolyII	66.79 ± 1.97	22.47 ± 2.41	8.60 ± 0.21	0.62 ± 0.03	1.52 ± 0.20	0.34
	PolyIII	66.02 ± 0.65	26.91 ± 0.43	6.76 ± 0.18	0.31 ± 0.04	0.00	0.41

**Table 5:** Distribution of hydrogen (%) based on the ^1^H NMR spectra of the biopolyols produced, grouped by chemical shift range.

Chemical Shifts/ppm	Proton Assignment	Distribution of Hydrogen/%		
		PolyI	PolyII	PolyIII
0.5–1.5	Aliphatics	63.2	55.7	35.9
1.5–3.0	Aliphatics α- to unsaturation or heteroatom	12.2	15.2	9.0
3.0–4.4	Alcohols	17.5	16.6	43.1
4.4–6.0	Methoxy, carbohydrates	2.8	4.5	6.6
6.0–8.5	Aromatics	4.3	8.0	5.4

**Table 7:** TGA analysis, with total weight lost and temperature of the DTG peak in each degradation step of the produced biopolyols, as well as the respective BioPUs.

Samples		Thermal Degradation Steps						Residue at 600 °C/wt.%
		1st Step		2nd Step		3rd Step		
		T_peak_/°C	Weight Lost/wt.%	T_peak_/°C	Weight Lost/wt.%	T_peak_/°C	Weight Lost/wt.%	
**Polyols**	PolyI	218	65	379	9	---	---	16
	PolyII	202	47	372	11	---	---	36
	PolyIII	268	16	395	36	---	---	39
**BioPU**	BPUI	213	18	304	28	427	30	23
	BPUII	215	18	288	26	422	32	19
	BPUIII	179	14	355	17	416	34	28

**Table 8:** Shore hardness, density, swelling rate and pull-off adhesion strength on carbon steel substrate of the synthesized BioPU.

BioPUs	Hardness/Sh D	Density/g cm^−3^	*SR*/%	Pull-Off Strength/MPa
BPUI	46 ± 4	1.09	4.2 ± 0.5	2.17 ± 0.40
BPUII	51 ± 2	1.16	6.4 ± 2.1	2.18 ± 0.13
BPUIII	65 ± 4	1.13	5.8 ± 0.75	0.84 ± 0.08

**Table 9:** Values of glass transition temperature (Tg), cross-linking density (*ν_x_*), and molecular weight of the chain between cross-linking points (*M_x_*), for the synthetized BioPU.

BioPUs	Tg/°C	*v_x_*/mol m^−3^	*M_x_*/Kg mol^−1^
BPUI	47	238	1.52
BPUII	61	383	1.01
BPUIII	77	1010	0.37

## Data Availability

The authors declare that data related to results reported in this paper is contained herein. Further data will be made available on request.
